# Rare metastasis of the urinary bladder from esophageal squamous cell carcinoma: a case report

**DOI:** 10.3389/fonc.2025.1515781

**Published:** 2025-06-19

**Authors:** Chunlei Zhang, Dehui Chang, Dongxing Wang, Jiale Zuo, Zhigang Cao

**Affiliations:** ^1^ Department of Urology, The 940 Hospital of Joint Logistics Support Force of Chinese People’s Liberation Amy (PLA), Lanzhou, Gansu, China; ^2^ Department of Clinical Medicine, Lanzhou University Second Hospital, Lanzhou, Gansu, China

**Keywords:** squamous cell carcinoma, esophagectomy, urinary bladder, bladder preservation, tumor markers

## Abstract

**Background:**

Squamous cell carcinoma (SCC) of the urinary bladder is rare, comprising less than 5% of all bladder cancers. There have been no previous reports of bladder SCC occurring as a metachronous metastatic tumor following curative resection for esophageal squamous carcinoma. This case report aims to address the challenges in treatment strategy posed by such occurrences by presenting a case of esophageal SCC with subsequent bladder metastasis.

**Case description:**

The patient is a 57-year-old Asian male with a 40-year history of heavy smoking. In July 2020, he was diagnosed with esophageal SCC, staged as cT2N1M0, and underwent radical surgery followed by adjuvant radiotherapy, chemotherapy, and immunotherapy. Post-surgery, he remained asymptomatic with regular check-ups showing no recurrence until November 2021, when he presented with hematuria. An MRU indicated a solid lesion in the bladder, and biopsy confirmed poorly differentiated SCC. A partial cystectomy was performed, followed by chemotherapy. Despite stable initial follow-ups, elevated CEA and SCC-Ag levels later suggested recurrence, confirmed by PET-CT and pathological examination showing lymph node metastases around the esophagus and in the neck. Subsequently, chemotherapy combined with immunotherapy was recommended.

**Conclusion:**

For solitary metachronous metastatic SCC of the bladder following esophageal SCC, surgical resection combined with postoperative adjuvant chemotherapy can be a viable treatment option. Partial cystectomy may be feasible for patients without lymphatic spread and who wish to preserve bladder function. Tumor markers CEA and SCC-Ag have proven effective in monitoring postoperative metastasis and can serve as reliable indicators for tumor follow-up.

## 1 Introduction

Squamous cell carcinoma (SCC) of the urinary bladder is histologically uncommon, accounting for less than 5% of all bladder cancers in the United States (1, 2). There have been no reports to date of it occurring as a metachronous metastatic tumor following curative resection for esophageal squamous carcinoma, which poses a significant challenge in choosing a treatment strategy for these patients. In this report, we present a case with a diagnosis of esophageal SCC with local lymph node metastasis for over four years and bladder metastasis for over two and a half years. The aim is to provide reference for treatment options for this patient population.

## 2 Case report

**Figure 1:**
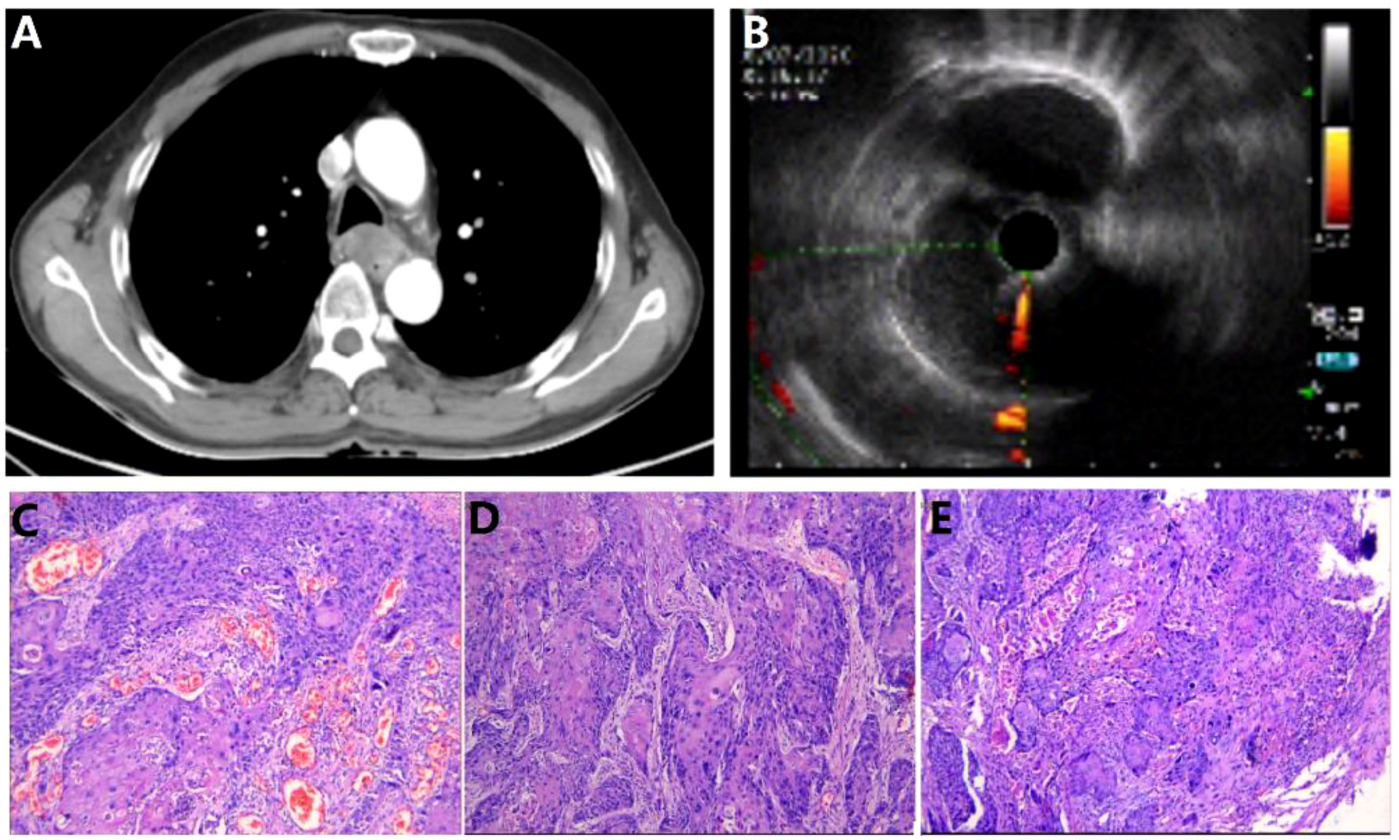
Esophageal cancer imaging report and pathology report. **(A)** Chest CT; **(B)** Endoscopic ultrasound (EUS). **(C–E)**. Postoperative esophageal cancer specimen pathology images.

This case report concerns a 57-year-old Asian male electrician with a notable 40-year history of heavy smoking, averaging 20 cigarettes daily, and no history of schistosomiasis. Despite this, he has minimal exposure to other potentially toxic substances and rarely consumes alcohol. His family history is clear of hereditary diseases, including cancer, and he has no personal history of infectious or parasitic conditions. The patient’s medical background is significant for benign colonic polyps, with no other reported health issues. He resides in a calm environment which contributes to his positive mental health, as reported by the patient himself. In July 2020, the patient presented with progressive dysphagia, decreased appetite, and weight loss, with no abnormalities observed in bowel movements or urination. Chest enhanced CT and esophageal ultrasound revealed esophageal cancer (**Figures 1A, B**). Clinical staging was determined as cT2N1M0. The patient underwent “thoracoscopic esophageal cancer radical surgery.” Pathological examination confirmed the diagnosis of moderately to well-differentiated squamous cell carcinoma (keratinizing type) involving the full thickness of the esophageal wall (**Figures 1C–E**). The tumor measured 5*3.5*1.5 cm and showed intravascular cancer embolism and neural involvement, with negative surgical margins. Lymph node examination revealed metastasis in the periesophageal lymph nodes (1/1) and recurrent laryngeal lymph nodes (1/1), while no cancer tissue was found in other lymph node groups. Immunohistochemical staining of the cancer cells showed CKP (+), CD34 (-), D2-40 (-), with 70% of the cells positive for Ki-67, PD-1 (-), PD-L1 (CPS <1). Immunohistochemical staining of the recurrent laryngeal lymph nodes indicated P63 (+), P16 (-), CKP (+), Villin (-), with 40% of the cells positive for Ki-67. After surgery, the patient received adjuvant radiotherapy (95% Planning Target Volume: 50Gy/1.8Gy), followed by chemotherapy (paclitaxel 400mg ivgtt on day 1 + cisplatin 30mg on day 1, 40mg on day 2-3, ivgtt, Q3W) combined with immunotherapy (Sintilimab 200mg ivgtt once a week) for six months.

**Figure 2:**
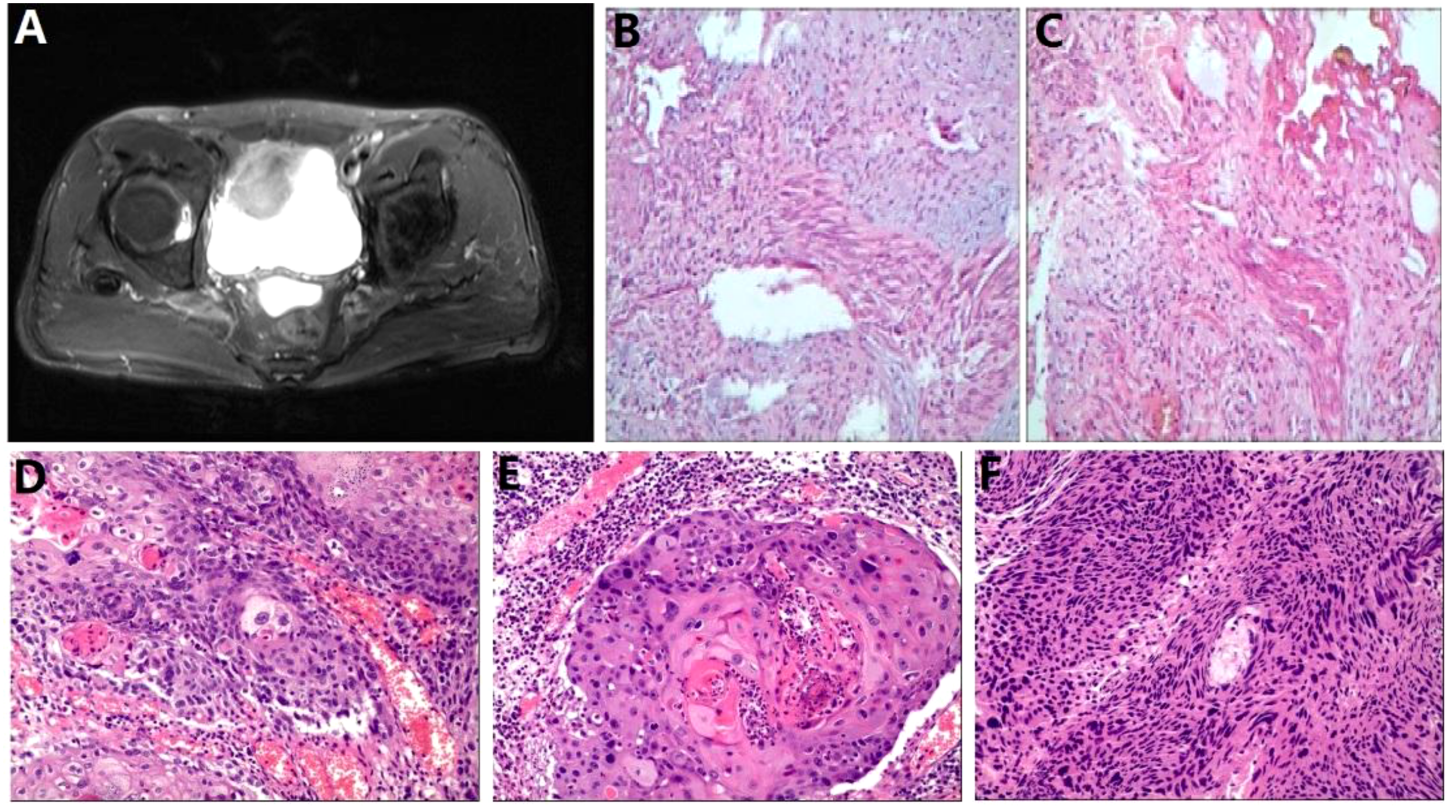
Bladder carcinoma imaging report and pathology report **(A)** Pelvic MRI; **(B, C)** Bladder tumor biopsy pathology images; **(D-F)** Postoperative bladder tumor specimen pathology images.

**Figure 3:**
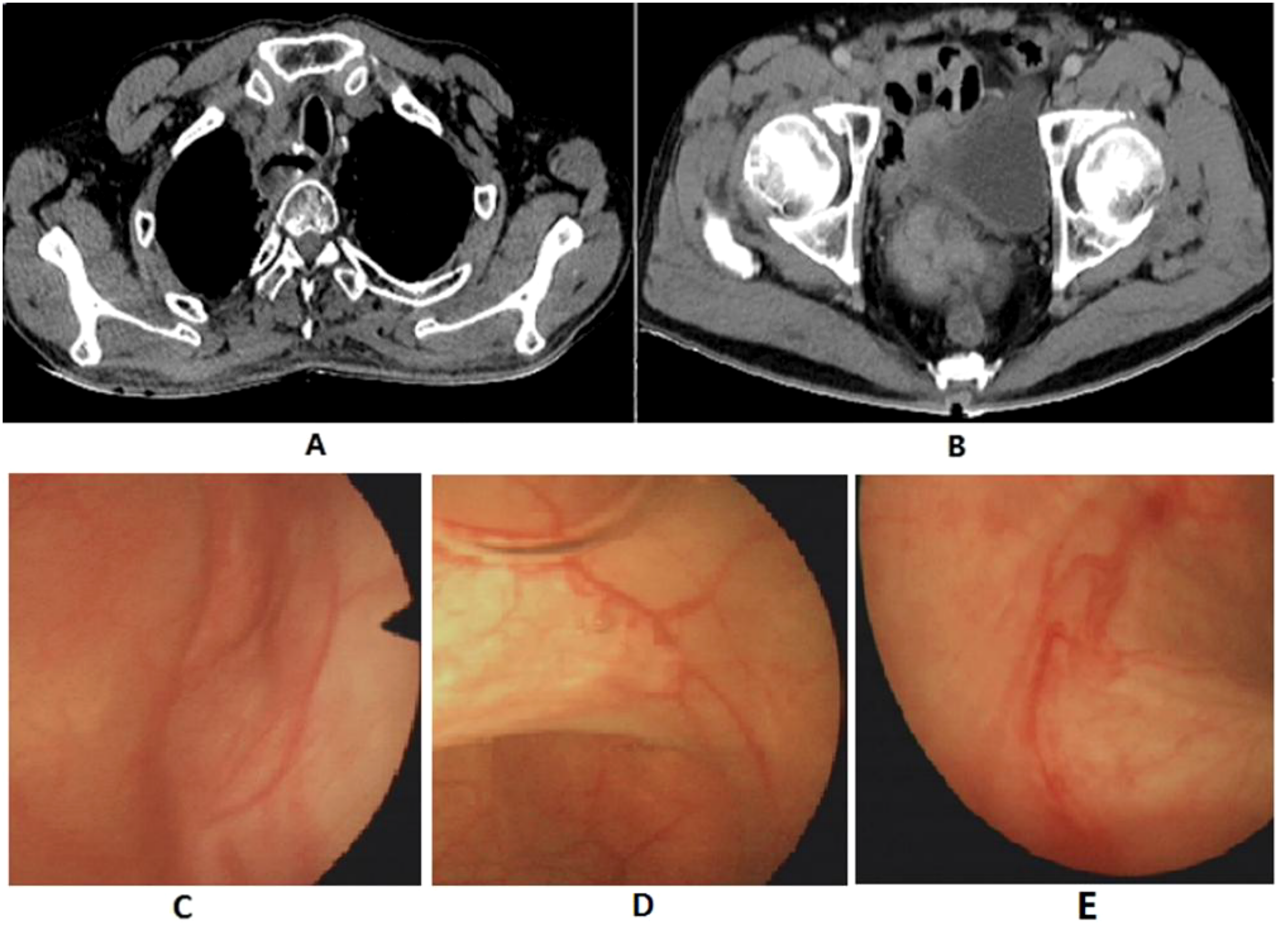
Postoperative follow-up imaging report. **(A)** Chest CT; **(B)** Pelvic CT; **(C-E)** Cystoscopy exam.

**Figure 4:**
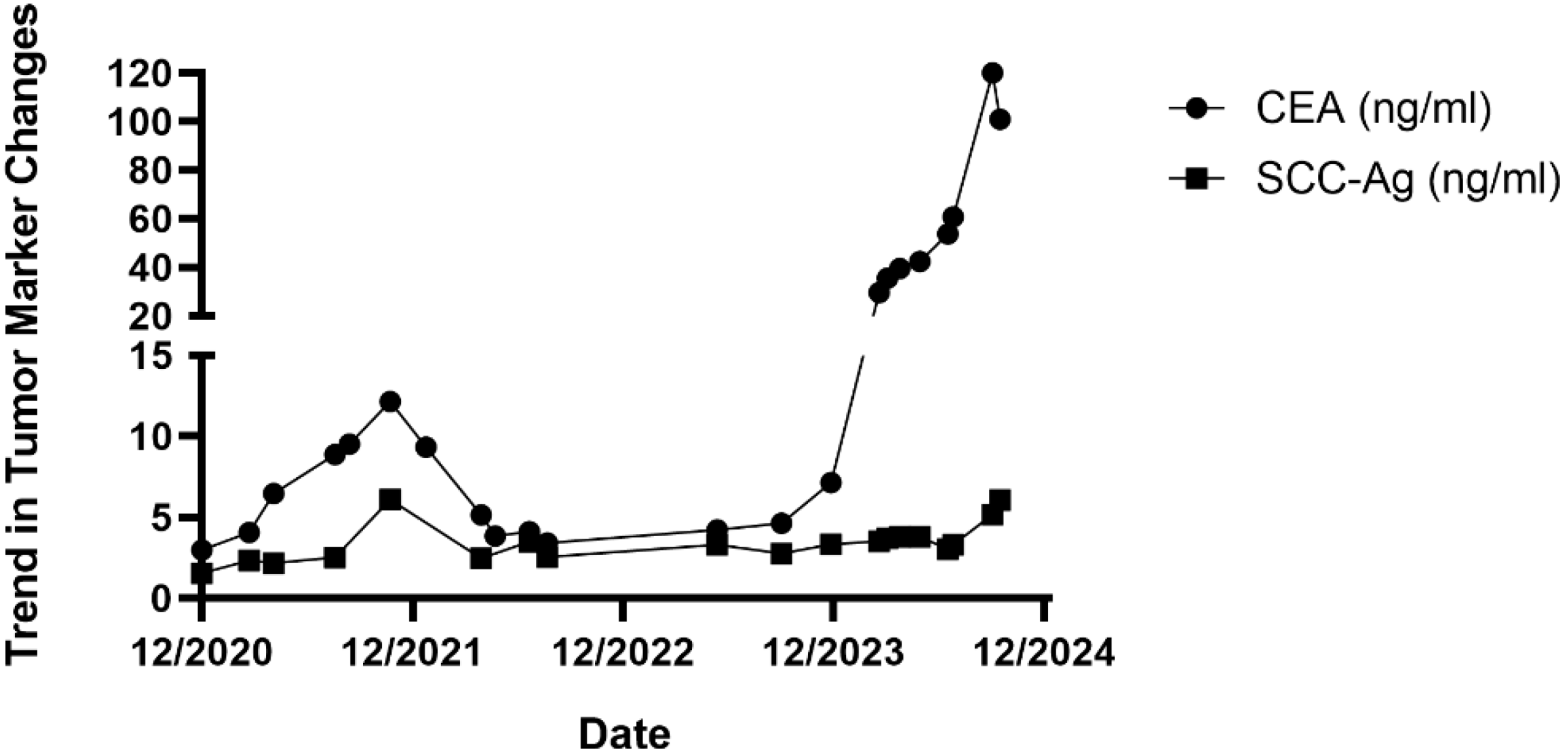
Temporal changes in serum tumor markers.

**Figure 5:**
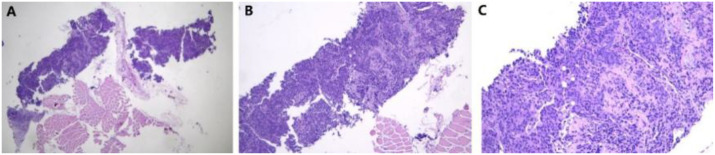
Pathology of fine needle aspiration biopsy of cervical lymph nodes. **(A)** 40×; **(B)** 100×; **(C)** 400×.

**Figure 6:**
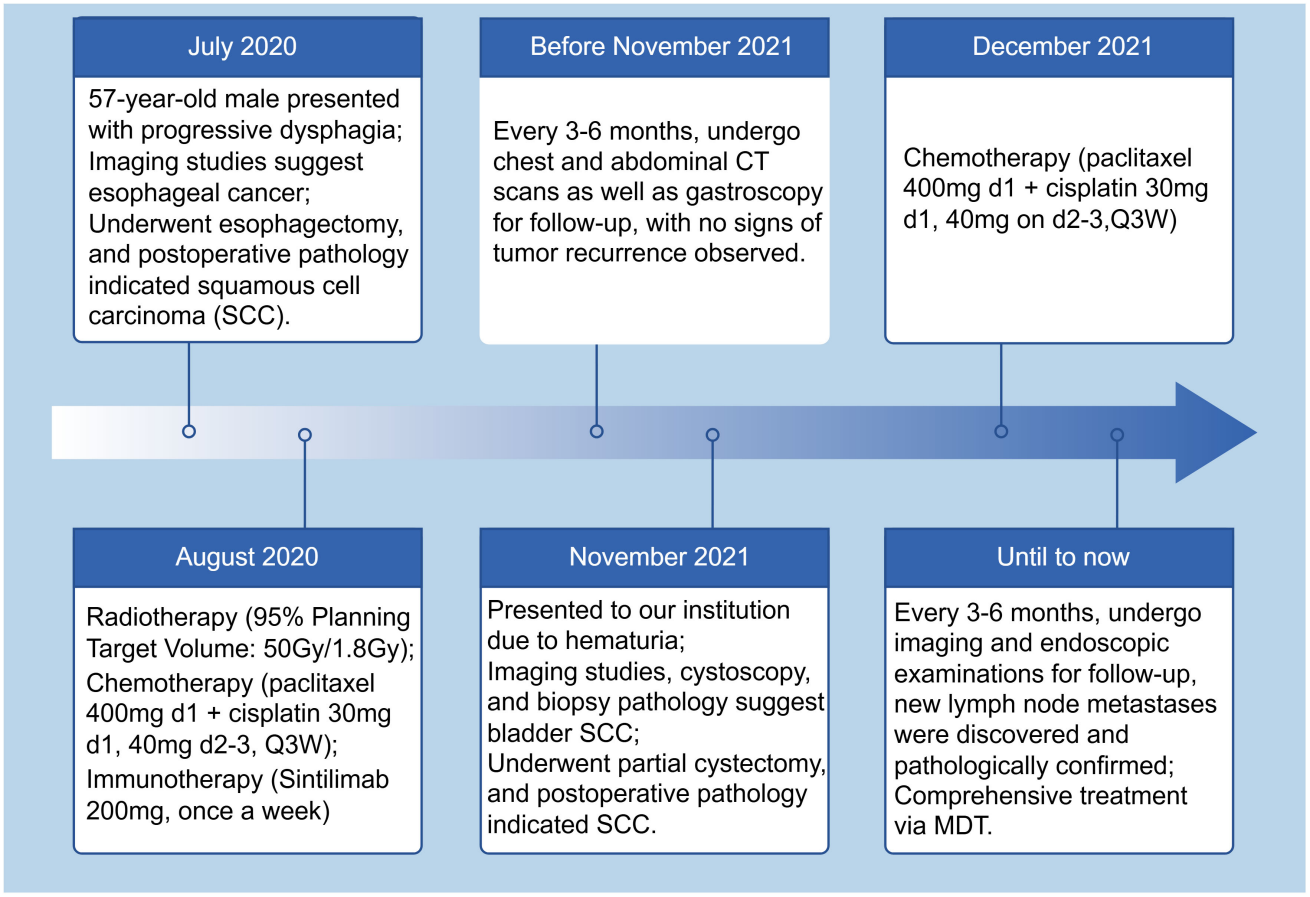
The patient’s timeline of consultations.

The patient quit smoking after undergoing curative surgery for esophageal cancer and maintains a normal diet and lifestyle with regular bowel movements and urination, without any significant symptoms of discomfort. Abdominal and thoracic CT scans, gastroscopy, as well as tumor markers tests [including carcinoembryonic antigen (CEA), and SCC Antigen (SCC-Ag)] are performed every 3 to 6 months, with no evidence of tumor recurrence or distant metastases found. However, in November 2021, the patient presented with symptoms of hematuria accompanied by a rise in tumor markers. And an MRU (Magnetic Resonance Urography) indicated a solid intramural lesion in the bladder (4.3*2.9 cm, **Figure 2A**). PET-CT (18F-FDG) indicated malignant tumor in the bladder, while no abnormal signals were observed at the gastroesophageal anastomosis or other sites in the body. Cystoscopy and biopsy indicate SCC (**Figures 2B,C**), urine cytology tests were negative. Due to the patient’s strong desire to preserve the bladder, a partial cystectomy was performed. Pathological examination revealed poorly differentiated carcinoma infiltrating the muscularis propria (**Figures 2D–F**). Immunohistochemical staining of the tumor cells showed CK5/6 (+), GATA3 (weak positive in a few cells), FOXA1 (-), CK7 (-), CK20 (-), Uroplakin II (-), HER2 (0, according to breast cancer criteria), with 60% of the cells positive for Ki-67. The cancer was considered to be SCC (keratinizing type), with no bladder *in situ* carcinoma component identified in the examined tissue. PD-1 (-), PD-L1 (CPS <1). The patient underwent four cycles of chemotherapy (paclitaxel 400mg ivgtt on day 1 + cisplatin 30mg ivgtt on day 1, 40mg on day 2-3, ivgtt, Q3W) after surgery. Postoperatively, the patient underwent follow-up examinations every 3 to 6 months, including chest CT, abdominal CT, gastroscopy, cystoscopy, and tumor markers which showed the condition to be stable. In March 2024, follow-up CT scans of the chest and pelvis showed no tumor recurrence (**Figures 3A, B**). The cystoscopic examination indicates that the wound inside the bladder is healing well and no new lesions are observed (**Figures 3C–E**). However, we discovered an elevated CEA level among the tumor markers (**Figure 4**), while SCC-Ag level remained relatively stable. This raised the possibility of tumor recurrence or metastasis. We recommended further PET-CT scans or adjuvant therapy to the patient. However, the patient declined our suggestions due to the absence of radiologically positive indicators at that time. During subsequent follow-ups, we observed a significant increase in both markers, starting in September (**Figure 4**). The patient then underwent a PET-CT scan, which indicated multiple lymph node metastases around the esophagus and in the neck, with no abnormalities detected in the bladder surgery area. Lymph node biopsy pathology suggested metastatic SCC, immunohistochemical staining of cancer cells: p40 (+), p63 (+), CKp (+), CK5/6 (Partial +), p53 (-, Nonsense Mutation), Ki67: 30% Positive (**Figures 5A–C**). These findings confirmed the presence of lymph node metastasis. After a Multi-Disciplinary Treatment (MDT) discussion, we recommended that the patient undergo chemotherapy combined with immunotherapy. The patient’s timeline of consultations is shown in **Figure 6** and he is expressed satisfaction with the treatment outcome.

## 3 Discussion

The most common sites of distant metastasis for esophageal SCC are the liver and lungs, with the bones, brain, and adrenal glands also being relatively common sites of spread (3). There have been a few reports of metastasis to other less common sites such as the kidneys (4, 5). In a retrospective study conducted by the Royal Hospitals Trust, a total of 282 cases of secondary bladder tumors were identified, accounting for 2.3% of all malignant bladder surgical specimens (6). The most common primary sites of tumors metastasizing to the bladder were the stomach (comprising 4.3% of all secondary bladder tumors), skin (3.9%), lungs (2.8%), and breast (2.5%) (6). However, to date, there have been no reports on esophageal cancer metastasizing to the bladder or bladder SCC as a metastatic cancer.

In oncology, the occurrence of multiple primary malignant tumors is not uncommon, with an incidence ranging from 0.52% to 11.7% (7). However, options for the treatment of multiple primary malignancies are quite limited. For both synchronous and metachronous malignancies, distinguishing between metastatic tumors and multiple primary malignancies is crucial, as it impacts treatment decisions and patient prognosis. For metastatic tumors, treatment decisions can be made according to the principles of post-metastasis therapy of the primary tumor. The treatment and prognosis for multiple primary tumors depend on the independent staging of each tumor (8, 9). Therefore, in this case report, identifying whether the bladder SCC is a primary or secondary tumor is crucial. For poorly differentiated SCC, it is still challenging from a pathological perspective to distinguish between primary and secondary tumors. Additionally, bladder SCC shares a high degree of overlap in the molecular immunohistochemistry profile with esophageal SCC. Therefore, after discussions among multiple pathology experts and considering that the microscopic morphology of the tumor cells and the immunohistochemical results are more consistent with the characteristics of metastatic squamous cell carcinoma, as well as the consistent expression of PD-1 and PD-L1 in both, this case report diagnoses the patient with metastatic bladder SCC.

SCC is one of the predominant pathological type of esophageal cancer. Patients experiencing recurrence or distant metastasis after curative treatment require systemic treatment plans; if metastatic lesions are resectable, surgical removal can be considered. Multiple reports confirm that metachronous tumor surgery following esophageal SCC can result in favorable prognosis (10–12). The first-line recommendation for systemic treatment includes immune checkpoint inhibitors (such as pembrolizumab, nivolumab, or durvalumab) combined with platinum-based chemotherapy using cisplatin and fluorouracil or with taxanes like paclitaxel (13). Bladder SCC accounts for approximately 2.5% of malignant bladder tumors, with a five-year survival rate of only 28% for muscle-invasive SCC (1, 2). The primary treatment currently is radical cystectomy combined with pelvic lymph node dissection, with local recurrence being the main cause of disease progression. Long-term prognosis has been a subject of debate. In a study of 16 cases of bladder SCC, of the 9 patients who underwent cystectomy, 5 achieved a five-year survival, while 4 died within two years. The remaining 7 patients who did not undergo cystectomy died at an average of 5.7 months (14). Additionally, a single-center statistical analysis conducted over 15 years involving 33 patients treated for pure bladder SCC showed a five-year survival rate of 33.3%, with survival rates correlating with tumor staging (15). These single-center case analyses reveal that five-year survival rates for bladder SCC are low and correlate with pathological tumor staging. However, patients who undergo radical surgery have significantly better outcomes than those who do not, highlighting the importance of surgical treatment in bladder SCC. At present, there is no solid evidence for the efficacy of neoadjuvant or adjuvant therapy; however, adjuvant radiotherapy can be considered for high-risk factors such as positive surgical margins (16), and some patients with advanced disease might consider combination chemotherapy with paclitaxel, ifosfamide, and cisplatin (17).

Given that we considered the bladder SCC to be metastatic at that time, and considering the stage of the patient’s bladder SCC and the strong desire for bladder preservation, as well as the fact that long-term survival can be achieved with the resection of distant metastatic lesions in esophageal cancer (13), a partial cystectomy was ultimately performed on the patient. Although the primary treatment for primary bladder SCC involves radical cystectomy combined with lymphadenectomy, some studies have indicated that part patients undergoing partial cystectomy can also achieve a degree of being disease-free (18). Regardless of whether the bladder tumor is a metachronous primary or a metastatic lesion, partial cystectomy combined with platinum-based chemotherapy can provide benefits to the patient. Therefore, we ultimately selected this treatment approach for the case. Two and a half years after undergoing partial bladder resection, new lymph node metastases were discovered around the esophagus and in the neck, while no recurrence or metastasis was observed at the bladder surgical site or in the surrounding lymph nodes. This confirms that these metastases indeed originate from esophageal SCC and suggests that bladder-preserving surgery may be a viable option for patients with bladder metastases originating from this type of cancer.

In diagnosing patients with advanced esophageal cancer, immunotherapy combined with chemotherapy is an effective first-line and second-line treatment option (19). Although this case shows negative PD-L1 expression, clinical trial results indicate that patients with low PD-L1 expression in advanced esophageal SCC can still benefit from immunotherapy combined with chemotherapy (20). The patient is currently in the late stage of metastatic esophageal cancer and is experiencing recurrence. Therefore, after multidisciplinary discussion, it is recommended that the patient proceed with chemotherapy in combination with immunotherapy. The patient initially received immunotherapy after the discovery of esophageal SCC with lymph node metastasis, which resulted in good tumor control. However, after discontinuing treatment, the patient experienced disease progression. This situation raises an important question: would maintaining immunotherapy post-surgery, or initiating it early upon the occurrence of bladder metastasis or the elevation of tumor markers, lead to better outcomes? This potential approach warrants further exploration. Although we recommended that the patient undergo immunotherapy following the partial cystectomy for bladder cancer, due to financial constraints, the patient only completed adjuvant chemotherapy with cisplatin and paclitaxel. It is crucial to consider the patient’s financial situation and insurance coverage when making treatment decisions, as these factors may limit the patient’s ability to access optimal therapy. Additionally, lymphatic metastasis of esophageal SCC tends to occur before hematogenous metastasis (21). While lymphatic spread was observed in the postoperative specimen of this case, during follow-up, distant metastasis developed ahead of recurrence and spread to the surrounding lymph nodes of the esophagus. It is also worth investigating whether the impact of immunotherapy combined with chemotherapy influences the metastatic biological characteristics of this disease.

There are no specific tumor markers for esophageal SCC. Identifying an effective tumor marker for monitoring tumor recurrence and metastasis in postoperative patients is of great significance for clinical treatment and disease monitoring. CEA is a glycoprotein involved in cell adhesion, which is highly expressed in the plasma of patients with various tumors and is commonly used as a tumor marker in clinical practice. In studies of tumor biomarkers for SCC, CEA has demonstrated good diagnostic value in oral SCC and lung adenocarcinoma (22, 23), and it can also predict the prognosis of patients after esophageal SCC surgery (24). SCC-Ag is a protein produced by SCC cells, and it is commonly used as a tumor marker for SCC (25). It is associated not only with the size of the tumor (26) and TNM staging (27) but can also serve as an effective predictive factor for therapeutic response (28). Additionally, it is related to treatment outcomes, including recurrence and prognosis (29). This indicates that SCC-Ag plays an active role throughout the diagnosis, treatment, and prognosis of SCC.

In this case report, CEA and SCC-Ag exhibited low levels in plasma postoperatively, and were significantly elevated in cases of bladder metastasis and lymphatic metastasis, with a significant decrease in SCC levels after tumor resection surgery, and a further decrease following chemotherapy. Notably, CEA levels began to rise significantly even before metastases were detected through CT imaging examinations. This indicates that both markers can serve as effective predictive factors of therapeutic response in this disease. The early predictive capability of CEA for metastasis is stronger, which can aid in early intervention and treatment before metastasis is clinically evident. When CEA levels begin to rise, it is recommended to conduct early PET-CT scans or pathological assessments to confirm the presence of any metastatic lesions.

## 4 Conclusion

In summary, for cases of solitary metachronous SCC of the bladder occurring after curative surgery for esophageal squamous carcinoma, surgical resection combined with postoperative adjuvant chemotherapy can be considered. Partial cystectomy appears to be a feasible treatment option for patients without radiologic evidence of lymphatic spread and who have a strong desire to preserve bladder function. The tumor markers CEA and SCC-Ag have a strong capability to monitor postoperative metastasis in esophageal SCC and serve as effective indicators for tumor follow-up monitoring.

## Data Availability

The original contributions presented in the study are included in the article/supplementary material. Further inquiries can be directed to the corresponding author.
